# M1 macrophage-derived exosomes synergistically enhance the anti- bladder cancer effect of gemcitabine

**DOI:** 10.18632/aging.204200

**Published:** 2022-08-03

**Authors:** Zhiling Tang, Chenye Tang, Chun Sun, Xiangjun Ying, Ruilin Shen

**Affiliations:** 1 Department of Urology Surgery, The Second Affiliated Hospital of Jiaxing University, Jiaxing 314001, China

**Keywords:** bladder cancer, macrophage, gemcitabine, exosome, drug loading

## Abstract

Gemcitabine (GEM) is one of the first choice drugs for treating bladder cancer. In this study, we loaded M1 macrophage-derived exosomes (M1-Exo) with GEM by ultrasonication technique to derive an M1-Exo-GEM drug delivery system, and then explored its effects on bladder cancer.

After inducing M1 polarization of macrophages *in vitro*, ultracentrifugation was performed to obtain M1-Exo, followed by construction of M1-Exo-GEM via ultrasonication technique. Mouse bladder cancer MB49 cells were chosen for study. CCK-8, PI staining and flow cytometry (FCM) assays were employed to assess the cell viability and apoptosis level. Inflammatory cytokines were detected by ELISA, while the protein expressions of Bcl-2, Bax and Caspase-3 were examined through Western-Blotting. After injecting M1-Exo-GEM into the tumor-bearing mouse model, the pathological changes were observed by H&E staining, the cancer cell damage was detected by TUNEL staining, and the apoptosis pathway activation was analyzed through immunohistochemical (IHC) staining and protein expression assays for Caspase-3 and Bax.

Our results showed that M1-Exo and GEM had cytotoxic effects on MB49 cells, which increased the apoptosis level and the inflammatory cytokine expressions. Compared to M1-Exo and GEM, M1-Exo-GEM was significantly more cytotoxic to MB49 cells while markedly up-regulating the expressions of inflammatory cytokines. In the tumor-bearing mouse model, M1-Exo-GEM significantly inhibited tumor growth and damaged tumor cells, which outperformed GEM. Meanwhile, it also increased the tissue levels of inflammatory cytokines.

This study finds that the drug delivery system composed of M1-Exo and GEM can act synergistically with GEM to exert cytotoxicity and induce inflammatory damage of bladder cancer cells.

## INTRODUCTION

Bladder cancer, as a common malignancy, currently has low sensitivity to radiotherapy and chemotherapy, which is characterized by strong invasiveness, as well as easy recurrence and metastasis. Gemcitabine (GEM) is a broad-spectrum antineoplastic agent that plays an important role currently as one of the major infusion chemotherapy drugs for bladder cancer in clinical practice [1]. In the cancer research, nano-scale drug carriers have gradually become the focus of treatment, which can deliver hydrophobic drugs, prolong the efficacy time and enhance the potency [2]. Liposomes, micelles, vesicles, etc. are all good carriers for delivering drugs [3]. As a hydrophobic drug, GEM has limited pharmacological availability, and its intravesical perfusion yields short efficacy duration and insufficient sustainability, which have become the treatment bottlenecks at present [4].

Macrophages are a kind of non-specific immune cells in the human body, which induce polarization through microenvironmental changes and produce specific functions, and their phenotypes represent the two extremes of their functional state [5]. Polarized macrophages can be roughly classified into two types: the classically activated M1 macrophages (pro-inflammatory macrophages) and the alternatively activated M2 macrophages (anti-inflammatory macrophages) [6]. The former highly expresses IL-12, NO and reactive oxygen species (ROS), which can dissolve tumor cells, and then produce immunostimulatory factors by presenting tumor-associated antigens to T cells, thereby promoting the proliferation of T and NK cells to enhance their anti-tumor effects [7]. The exosomes derived from M1 macrophages also have anti-tumor effects, which can thus be used synergistically with antineoplastics [8]. In this regard, this study uses the M1 macrophage-derived exosomes (M1-Exo) to construct a drug delivery system of GEM for bladder cancer.

## MATERIALS AND METHODS

### Construction and characterization of M1-Exo-GEM system

Mouse mononuclear macrophages RAW264.7 (Procell Biotechnology, Wuhan, China) were thawed and cultured in a complete medium at 37° C and saturated humidity with 5% CO_2_. Then, logarithmic phase cells were seeded onto 6-well plates at 10^6^/well and adaptively cultured for 24 h, followed by induction of M1 polarization. RAW264.7 cells were treated with 200 ng/ml PMA (Sigma, USA) for 6 h, and then added with 1 μg/ml LPS (Sigma, USA) and 20 ng/ml IFN-γ (Sigma, USA) for induction and cultivation for 48 h [9]. The macrophages were divided into M0 and M1 groups. FCM was employed to determine the proportion of M1 cells (F4/80+CD86+), while ELISA kit (Nanjing Jiancheng Bioengineering Institute, Nanjing, China) was utilized to examine the levels of M1 marker cytokines TNF-α, IL-1β and IL-6. After successful induction of M1 macrophages, the medium was replaced with complete Exo serum-free DMEM (Gibco, USA), and the cell cultivation was accomplished in a flask for 48 h. Afterwards, the cells and medium were collected, and Exos were isolated through ultracentrifugation as per the instructions of an exosome extraction kit (SBI, USA). The Exos from M1 macrophages were defined as M1-Exo.

For construction of M1-Exo-GEM, we adopted the ultrasonication technique [10]. M1-Exos solution with a 1 mg/mL protein concentration was prepared, added with GEM and then ultrasonicated in 6 cycles of 30 s each time at a 20 W power. After each cycle, the M1-Exo-GEM system was placed on ice for 2 min of rest. Following ultrasonication, the M1-Exo-GEM system was incubated at 37° C for 60 min, and the Exo membranes were restored. Centrifugation was performed at 10,000 g to remove excess GEM, and M1-Exo-GEM was enriched.

### Dynamic light scattering (DLS) determination of M1-Exo-GEM particle size

Each 0.5 mL of M1-Exo-GEM mother solution was diluted and mixed well in 4.5 mL of ultrapure water. After passing through a 0.22 μm filter, particle size was determined with a Nano-ZS90 Zetasizer (Malvern, UK), followed by recording and report generation.

### Nanoparticle tracing analysis (NTA) of Exo particle size distribution

The M1-Exo-GEM mother solution was diluted to 10 mL with water at a ratio of 1:7500, and then injected into the Zetasizer by setting parameters. Particle size distribution was analyzed, followed by recording and report generation.

### Morphological analysis of M1-Exo-GEM

The M1-Exo-GEM was diluted with Exo-free PBS, resuspended and mixed well. Then, 20 μL was taken, Exos were dropped onto copper grids, and 1 min later, the samples were blotted with filter paper. One min after dropwise addition of 1% uranyl acetate, the samples were blotted with filter paper, dried under an incandescent lamp, and morphologically observed with a transmission electron microscope (TEM), followed by imaging and recording. Western-Blot was performed to assess the expressions of Exo marker proteins CD63, CD81 and ALIX [11].

### HPLC determination of GEM loading capacity

HPLC conditions: Agilent 1260-G7115A HPLC system (Agilent, USA): 1260 pump and G7115A DAD detector; column: Ultimate XB-C18 column (4.6 mm × 250 mm, 5 μm); mobile phase: acetonitrile: 0.1% formic acid (50:40, v/v); column temperature: 30° C; flow rate: 1.0 mL/min; detection wavelength: 312 nm; and injection volume: 20 μL.

### Standard curve

GEM solutions with concentrations of 1, 5, 10, 20, 40 and 50 μg/mL were separately prepared, and their peak areas were measured at 312 nm by HPLC, followed by plotting of a standard curve.

### Drug loading capacity

An appropriate amount of the prepared M1-Exo-GEM solution was centrifuged at 15,000 rpm for 15 min. Although the M1-Exo-GEM did not precipitate, the free GEM crystals precipitated. The supernatant was collected and demulsified with the mobile phase, followed by HPLC measurement of peak area at 312 nm. Finally, the GEM loading capacity was calculated based on its standard curve.

### Stability

For stability investigation, we placed the PTX-M1-Exos system separately at 4° C and room temperature, and examined the changes in particle size daily for 7 d.

### Effects of M1-Exo-GEM on MB49 cells

Mouse bladder cancer MB49 cells were placed in complete RPMI-1640 medium containing 10% fetal bovine serum (FBS), 100 IU/mL penicillin and 100 IU/mL streptomycin, and routinely cultured in a sterile incubator maintained at 37° C with 5% CO_2_. The medium was replaced regularly, and the cells were passaged until 80–90% confluence. The passaged cells were then further cultured routinely. When the confluence reached 80–90% again, the cells were collected or tested. The experimental cells must be in the logarithmic phase. We divided the cells into the Control, GEM, M1-Exo and M1-Exo-GEM groups. The Control group was not added with drug or Exos. The GEM group consisted of cells treated with 7.5 μg/ml GEM; the M1-Exo group comprised 40 μg/ml (protein content) M1-Exo-treated cells; and the M1-Exo-GEM group comprised cells treated with 7.5 μg/ml GEM and 40 μg/ml M1-Exo.

### CCK-8 assay

MB49 cells were seeded onto 96-well plates, with three replicate wells per group, and cultured in a 37° C, 5% CO_2_ incubator. Cell viability was assayed separately at 12 h, 24 h and 48h. After replacing the medium with serum-free one, 10 μl of CCK-8 reagent (Beyotime Biotechnology, Shanghai, China) was added for staining, and then the incubation continued for an additional 4 h, followed by measurement of 450 nm absorbance with a microplate reader. Cell viability was calculated against blank medium, and the results were presented in %.

### FCM

MB49 cells were seeded onto 6-well plates, treated with GEM and M1-Exo-GEM for 24 h, and then the suspended cells were collected. The adherent cells were digested with 0.25% trypsin, washed 2–3 times in PBS, and centrifuged at 2,000 rpm for 10 min. Next, the cells were collected, resuspended in pre-cooled PBS, centrifuged again, and then resuspended in binding buffer and stained with 5 μl of Annexin V-FITC (BD Biosciences, USA) in the dark for 15 min. Thereafter, the cells were stained with 5 μl of PI (BD Biosciences, USA) for 5 min, adjusted to a 500-μl volume and analyzed by FCM.

### Enzyme-linked immunosorbent assay (ELISA)

For assessment of inflammatory cytokine expressions, the MB49 cells were seeded onto 12-well plates, and treated with GEM and M1-Exo-GEM for 24 h. Then, the medium was collected, and centrifuged at 10,000 g to remove suspended cells and debris. Finally, the supernatant was collected for assaying the expressions of inflammatory cytokines (mainly IL-1β, TNF-α and IL-6) as per the instructions of ELISA kit (Nanjing Jiancheng Bioengineering Institute, Nanjing, China). The results were presented in terms of ng/ml.

### Immunofluorescent (IFC) staining

MB49 cells were seeded onto the coverslips, treated with GEM and M1-Exo-GEM for 24 h, fixed in 4% formaldehyde at room temperature for 0.5 h, permeabilized with 0.2% Triton X-100 for 5 min, and then examined for the expressions of Caspase-3 and Bax. The next step was overnight incubation at 4° C with TBNT dilution of monoclonal antibody (Abcam, USA). Afterwards, the cells were washed twice with PBS, incubated with fluorescent secondary antibody, and then mounted with 95% glycerol and observed under a fluorescence microscope.

### PI staining

MB49 cells were seeded onto 12-well plates, and treated with GEM and M1-Exo-GEM for 24 h. After discarding the medium, the cells were washed twice with PBS, and then stained with 1 μg/ml PI reagent (Beyotime Biotechnology, Shanghai, China) for 30 min, followed by twice washing with PBS. Positive cells were indicated by red fluorescence.

### Western blotting

For expression level determination of apoptosis-related proteins Caspase-3, Bcl-2, Bax, cells were treated with GEM and M1-Exo-GEM for 24 h, lysed on ice with 1.0 ml of RIPA lysate (Beyotime Biotechnology, Shanghai, China) for 30 min, and then centrifuged at 10,000 g for 15 min, followed by collection of supernatant for protein quantification. Next, SDS-PAGE gel electrophoresis was performed at 80V–120 V, and membranes were transferred at a constant 300 mA for 0.5–2 h. The PVDF membranes were blocked with 5% skimmed milk powder for 2 h, and incubated overnight at 4° C with Caspase-3, Bcl-2 and Bax monoclonal primary antibodies (1:500 dilutions with TBST), and then with horseradish peroxidase (HRP)-labeled goat anti-rabbit secondary antibody (1:2000 dilution; Abcam, USA). Afterwards, chemiluminescent immunoassay was performed, and the optical density (OD) was analyzed via Image Pro-Plus 6.0 software. The results were expressed as OD comparisons between the target proteins and the internal reference (GAPDH).

### PKH-67 labeling of cellular uptake of Exos

Cells were seeded onto the slides, and after adherence, PKH-67 (1:100 dilution; Abcam, USA) was dropped onto the slides. After completion of incubation, the slides were washed 3 times with PBS, added with anti-fluorescence quenching agent, and then observed and photographed under a fluorescence microscope.

### M1-Exo-GEM intervention in tumor-bearing mice

The present animal experimentation was in line with the ethical norms. Animal breeding and experiments conformed to the relevant animal welfare regulations, and the experimental protocol was reviewed and approved by the Ethics Committee of Jiaxing University.

To investigate the anti-tumor effect *in vivo*, the MB49 tumor-bearing mouse model was given tail vein injection. Healthy 5–6-weeks-old female BalB/c mice weighing about 20 g were inoculated subcutaneously with 1×10^6^ MB49 cells per mouse at the right anterior armpit, and the tumor volume was measured at 3-d intervals. Tumor volume (mm^3^) = abcπ/6, where a denotes the longest tumor diameter, b denotes the shortest tumor diameter, and c is the tumor spherical height. After the tumor volume reached 50–100 mm^3^, the tumor-bearing mice were randomized into 4 groups (n= 10), namely Control, GEM, M1-Exo and M1-Exo-GEM. Tail vein injection was given such that the GEM dose in the GEM and M1-Exo-GEM groups was 5 mg/kg. The initial administration was recorded as day 0, and the administration was given once every 3 days for a total of 4 times. Attention was paid to observing the state of each mouse. Their body weights were weighed every 3 days, the tumor volumes were measured, and the tumor growth curves were drawn. After the treatment cycle, the mice were sacrificed by cervical dislocation, and then the solid tumors were harvested and stored at -80° C for subsequent use.

### H&E staining

The tumors were embedded in paraffin and sectioned. The tissue sections were baked at 45° C for 2 h, and then treated with gradient concentrations of xylene and ethanol. After washing with water, the tissue sections were stained with hematoxylin for 10 min, washed with tap water and then treated with 1% hydrochloric acid ethanol. Following dehydration of ethanol, the tissue sections were stained with eosin solution, dehydrated, permeabilized, and then mounted with neutral gum and observed under a light microscope.

### TUNEL staining

The tissues were pretreated by the same procedure as H&E staining. Then, the sections were incubated in an incubator with proteinase K for 30 min. After washing with PBS, lysis buffer was added to break the cell membranes. The TdT and dUTP in TUNEL staining kit were mixed at a 1:9 ratio, and dropped onto the sections. The incubation continued for an additional 2 h, which was followed by nuclear counterstaining. Finally, the counts of TUNEL-positive cells were observed microscopically.

### Immunohistochemical (IHC) staining

The tumor tissues were fixed with 4% paraformaldehyde, embedded in paraffin and sectioned. The tissue sections were soaked in 1:50 acetone solution for 3 min and then dried, followed by treatment with xylene and absolute ethanol. Next, antigen retrieval was performed at 92–98° C in 0.01 mol/L citrate buffer. The next step was treatment with 3% hydrogen peroxide for 10–15min to remove endogenous peroxidase activity, and a subsequent blocking with 5% BSA at 37° C for 15–30 min. Afterwards, the tissue sections were incubated with YAP and TAZ monoclonal antibodies (Abcam, USA) at 37° C for 1–2 h, and then with HRP-labeled avidin at 37° C for 20 min, followed by staining with DAB reagent for 3–5 min. Finally, the tissue sections were counterstained with hematoxylin, dehydrated, permeabilized, and then mounted in resin.

### PKH-67 IFC staining

Exos were labeled with PKH-67, whose level of enrichment in tumor cells was detected.

### Western blotting

The tumor tissues were ground with liquid nitrogen, and lysed with 1.0 ml of RIPA buffer to extract the proteins, followed by detection of Caspase-3, Bcl-2 and Bax in cells as per the Western blotting procedure.

### Statistical methods

SPSS 17.0 was used for statistical analysis and processing, and all measurement data were presented as $\left(\bar{x}\pm \text{s}\right)$. After homogeneity test of variances, the two independent samples t-test was employed to perform analysis between two sets of data, while the one-way ANOVA was used between three and more sets of data. The subsequent pairwise comparison between groups was made by LSD method. All the foregoing tests were two-sided, and the differences were considered statistically significant when P< 0.05.

### Data availability statement

The data that support the findings of this study are available from the corresponding author upon reasonable request.

## RESULTS

### Construction and characterization of M1-Exo-GEM

**Figure 1:**
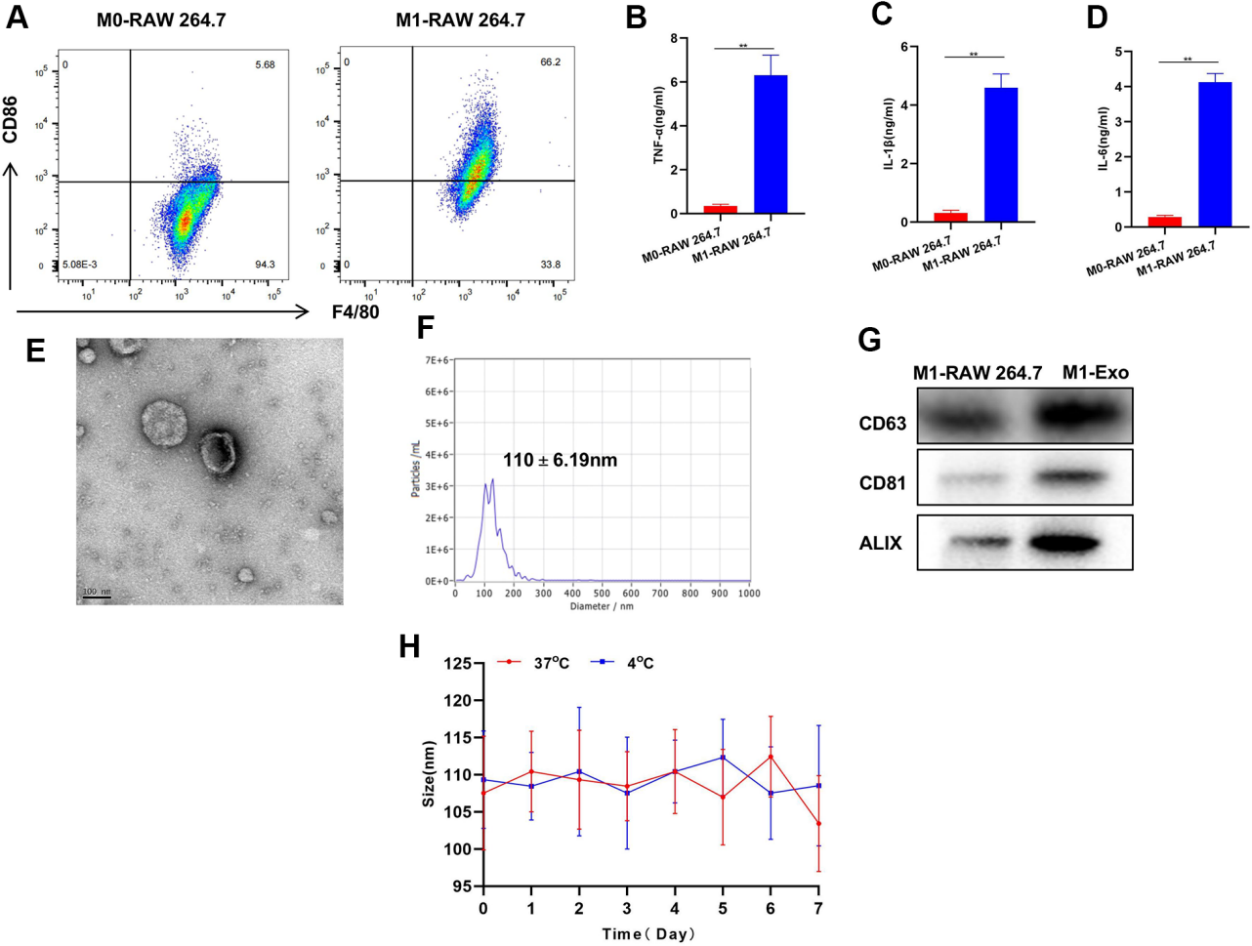
**Construction and characterization of M1-Exo-GEM.** (**A**) After induction, M0 macrophages were successfully polarized into M1 phenotype. (**B**–**D**) Expressions of M1 macrophage markers TNF-α, IL-6 and IL-1β were significantly up-regulated, which were higher than those in M0 macrophages, showing significant inter-group differences, **P<0.05. (**E**–**G**) M1-Exo-GEM had a circular pie appearance, with a particle size of around 100 nm. Meanwhile, the up-regulated expressions of marker proteins indicated that the Exos were morphologically intact, which conformed to relevant characteristics. (**H**) Investigation of Exo stability revealed insignificant changes in particle size of M1-Exo-GEM at 4° C and 37° C within 1 week of measurement.

We examined the induction of M1 macrophages and found that LPS and IFN-γ could successfully induce the polarization of M0-RAW 264.7 to M1 phenotype. Meanwhile, the levels of M1 cell markers TNF-α, IL-6 and IL-1β were significantly elevated (Figure 1A–1D). This suggests successful induction of M1 macrophages. M1-Exo-GEM retained the Exo morphology after GEM was complicated by ultrasonication, which looked like a circular pie (Figure 1E). Besides, the particle size of Exos was around 100 nm, which conformed to the morphological characteristics of Exos (Figure 1F). Detection of Exo markers revealed that CD63, CD81 and ALIX were up-regulated in M1-Exo-GEM (Figure 1G).

During the GEM loading characterization, we found that 1 mg of M1-Exo-GEM contained 15 μg of GEM. The particle size of M1-Exo-GEM changed little within 1 week of measurement regardless of whether the temperature was 4° C or 37° C, implying that M1-Exo-GEM could exist stably within that 1 week period. Accordingly, it can be inferred that M1-Exo-GEM can exist stably during the biological circulation process, which will not release GEM prematurely due to carrier rupture (Figure 1H).

### Effect and mechanism of M1-Exo-GEM on MB49 cytotoxicity

**Figure 2:**
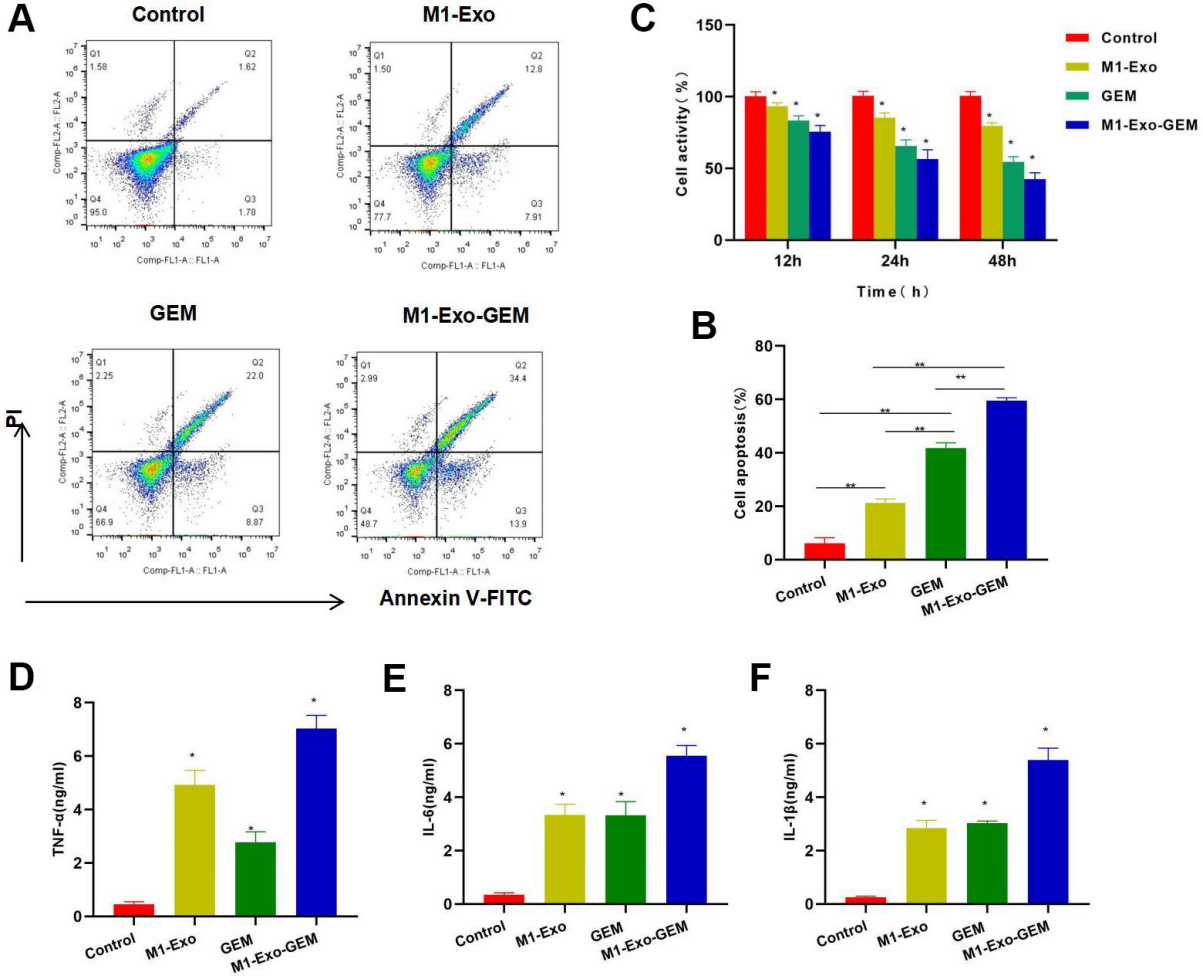
**Cytotoxicity of M1-Exo-GEM against MB49.** (**A**, **B**) FCM found that M1-Exo and GEM could induce apoptosis, and M1-Exo-GEM could further increase the apoptosis rate significantly than M1-Exo and GEM, **P<0.05. (**C**) Cell viability assay revealed that M1-Exo and GEM could inhibit cell viability in a time-dependent manner. In M1-Exo-GEM group, the cell viability was further reduced, which was lower than that in M1-Exo and GEM groups, *P<0.05 vs. Control group. (**D**–**F**) In the inflammatory cytokine detection, M1-Exo and GEM could promote the expressions of TNF-α, IL-6 and IL-1β, as manifested by significantly higher levels than those in the Control group, while the M1-Exo-GEM group exhibited higher levels of inflammatory cytokines than those in the M1-Exo and GEM groups. *P<0.05 vs. Control group.

FCM found that M1-Exo or GEM alone had prominent toxicity to MB49 cells, showing significantly higher apoptosis rates than the Control group, while M1-Exo-GEM was significantly more cytotoxic than M1-Exo and GEM (Figure 2A, 2B). Cell viability assay also demonstrated that M1-Exo-GEM could reduce cell viability, which outperformed M1-Exo and GEM (Figure 2C). In the inflammatory cytokine detection, M1-Exo and GEM could promote the expressions of TNF-α, IL-6 and IL-1β, as manifested by significantly higher levels than those in the Control group, while the inflammatory cytokine levels in M1-Exo-GEM group were higher than those in the M1-Exo and GEM groups (Figure 2D–2F).

### Effect of M1-Exo-GEM on MB49 apoptosis pathway

**Figure 3:**
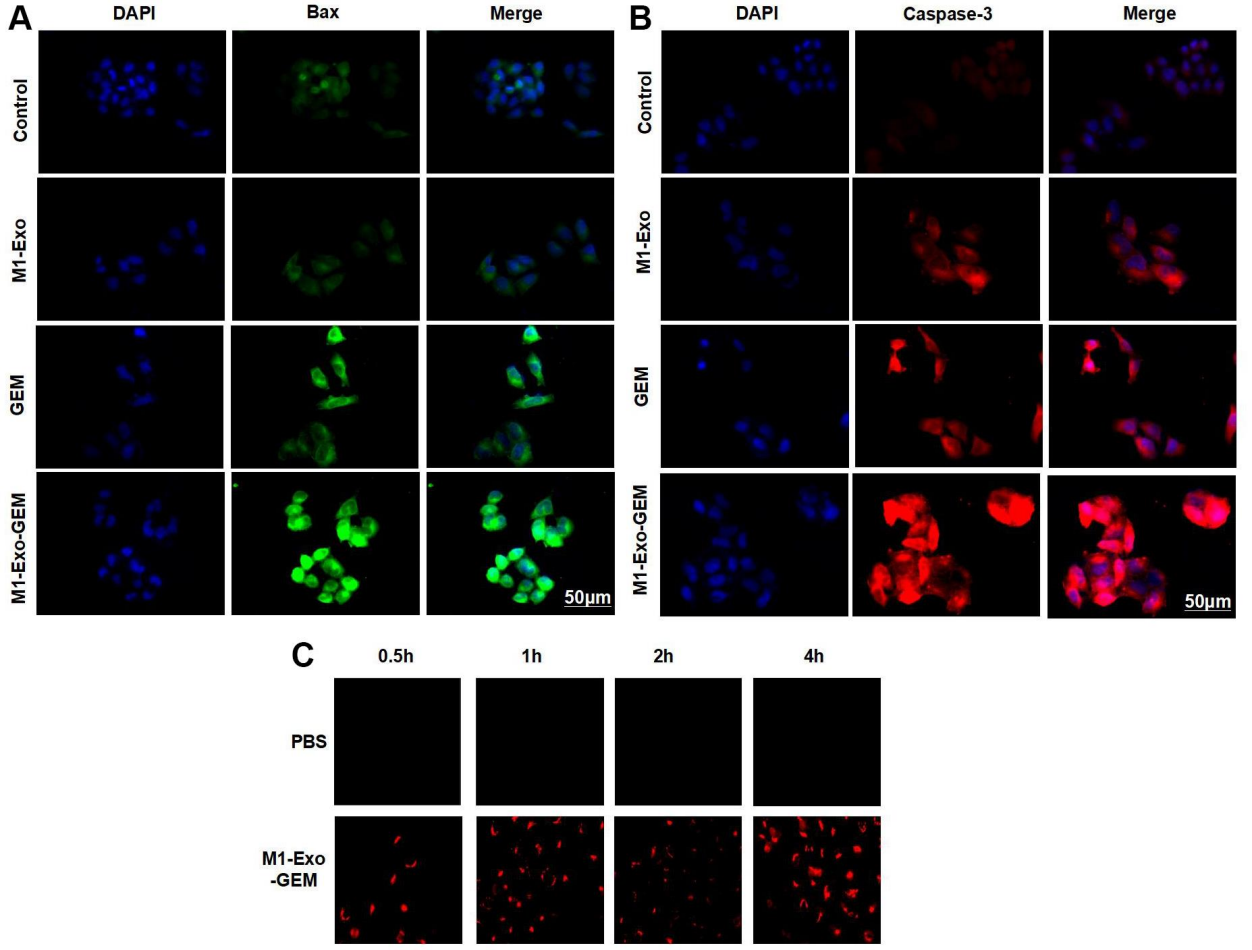
**Effect of M1-Exo-GEM on MB49 apoptosis.** (**A**, **B**) Caspase-3 and Bax were negatively expressed in the Control group, while M1-Exo and GEM could enhance the Caspase-3 and Bax expressions, as manifested by significantly higher fluorescence intensity than that of Control. In the M1-Exo-GEM group, these expressions were further up-regulated, with higher fluorescence intensity than that of M1-Exo and GEM. (**C**) M1-Exo-GEM could be taken up by MB49 cells, which were PKH-67-positive.

**Figure 4:**
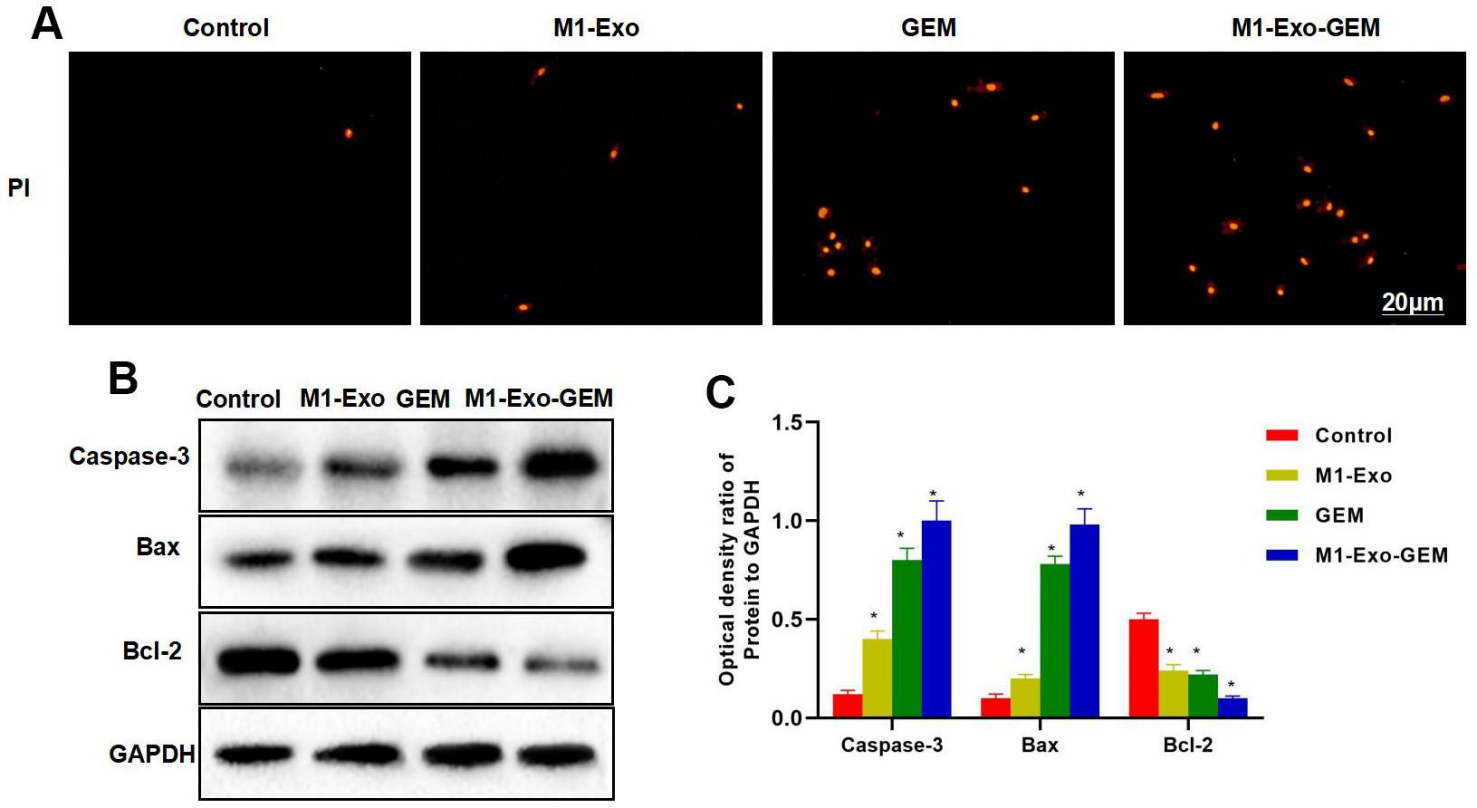
**Effect of M1-Exo-GEM on MB49 apoptosis.** (**A**) In PI staining, M1-Exo and GEM could promote apoptosis, as manifested by significantly higher number of positive cells than that of Control group, while M1-Exo-GEM could further promote apoptosis. (**B**, **C**) According to protein assay results, Bcl-2 was highly expressed in the Control group, while Caspase-3 and Bax were lowly expressed. GEM and M1-Exo enhanced the expressions of Caspase-3 and Bax and inhibited the Bcl-2 expression, suggesting their pro-apoptosis effect. M1-Exo-GEM could further promote the apoptosis signal activation. *P<0.05 vs. Control group.

Our IFC staining assay revealed that the Caspase-3 and Bax were negatively expressed in Control, while M1-Exo and GEM could promote the expressions of Caspase-3 and Bax, as manifested by significantly higher fluorescence intensity than that of Control. In M1-Exo-GEM group, these expressions were further up-regulated, with higher fluorescence intensity than that of M1-Exo and GEM (Figure 3A, 3B). We used PKH-67 to trace and label Exos, and found cellular enrichment of M1-Exo-GEM after 0.5 h, and an excellent Exo uptake capacity of MB49 cells (Figure 3C). PI staining also revealed that M1-Exo and GEM could promote apoptosis, as manifested by significantly higher number of positive cells than that of Control, while M1-Exo-GEM could further promote apoptosis (Figure 4A). According to protein assay results, GEM and M1-Exo also enhanced the expressions of Caspase-3 and Bax and inhibited the Bcl-2 expression, suggesting their pro-apoptosis effect. M1-Exo-GEM could further promote the apoptosis signal activation (Figure 4B, 4C).

### Effect of M1-Exo-GEM on tumor-bearing mice

**Figure 5:**
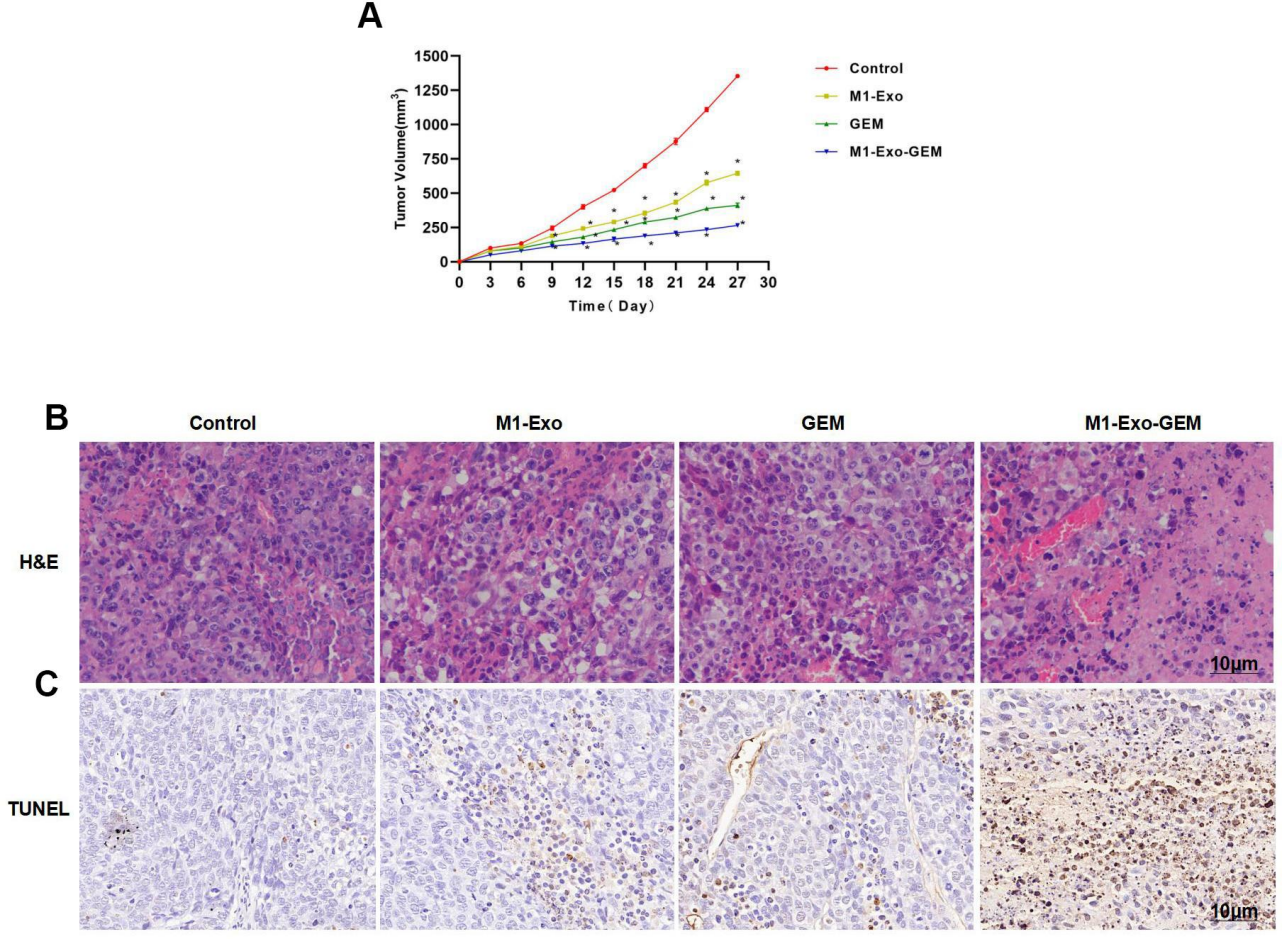
**Effect of M1-Exo-GEM on tumor-bearing mice.** (**A**) M1-Exo-GEM could inhibit the tumor growth, as manifested by significantly lower tumor volume than that in the M1-Exo and GEM groups. *P<0.05 vs. Control group. (**B**) In H&E staining, obvious inflammation and edema-like lesions appeared in the M1-Exo and GEM groups, as well as damage of tissues. In the M1-Exo-GEM group, tissue necrosis occurred, and tumor cells were obviously damaged. (**C**) In TUNEL staining, no apparently damaged cells were found in the Control group, which were TUNEL-negative. Obvious cell damage occurred in the M1-Exo and GEM groups. M1-Exo-GEM group exhibited significantly higher number of TUNEL-positive cells than that in the M1-Exo and GEM groups.

In mouse model, we found that M1-Exo and GEM could inhibit the tumor growth. In the tumor growth curves, the tumor volumes of M1-Exo and GEM groups were lower than the Control group over time. M1-Exo-GEM could further inhibit the tumor growth, where the tumor volume was lower than the M1-Exo and GEM groups (Figure 5A, 5B). H&E staining demonstrated that the tumor tissues in the Control group had no obvious cell damage or inflammation or edema. Contrastively, evident inflammation and edema occurred in M1-Exo and GEM groups, showing significant differences from the Control group. The cellular and tissue damages were further exacerbated in the M1-Exo-GEM group, with appearance of necrosis-like changes. M1-Exo-GEM exhibited stronger tumor cytotoxicity than M1-Exo and GEM (Figure 5C). TUNEL staining also revealed significantly higher number of TUNEL-positive cells in the M1-Exo-GEM group than that in the M1-Exo and GEM groups (Figure 5).

### Effect of M1-Exo-GEM on tumor apoptosis in tumor-bearing mice

**Figure 6:**
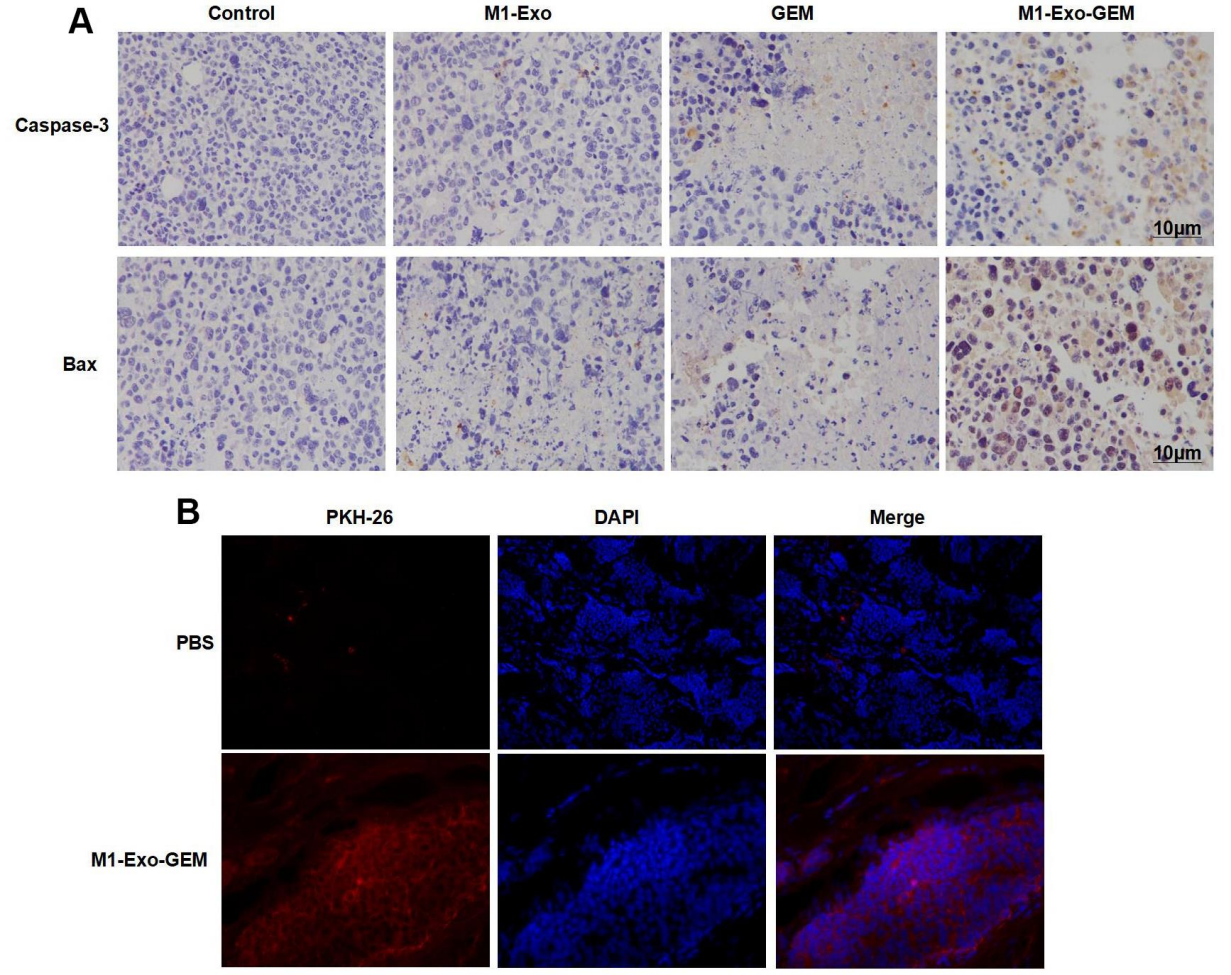
**Effect of M1-Exo-GEM on tumor apoptosis in tumor-bearing mice.** (**A**) In IHC staining, Caspase-3 and Bax were negatively expressed in the Control group. In the M1-Exo and GEM groups, these expressions were significantly up-regulated, where pathological changes of tissue damage occurred. In the M1-Exo-GEM group, Caspase-3 and Bax expressions were significantly up-regulated, showing higher levels than those in the M1-Exo and GEM groups. (**B**) PHK-67 tracing of Exos revealed enrichment of M1-Exo-GEM in tumor tissues, indicating that tumor tissues could take up Exos.

We examined the Caspase-3 and Bax expressions in tumor tissues. After IHC staining, Caspase-3 and Bax were negatively expressed in the Control group. M1-Exo and GEM groups exhibited significantly up-regulated Caspase-3 and Bax expressions, where pathological changes of tissue damage occurred. In the M1-Exo-GEM group, Caspase-3 and Bax expressions were significantly up-regulated, showing higher levels than those in the M1-Exo and GEM groups (Figure 6A). Further, PKH-67 labeling of Exos found that M1-Exo-GEM was enriched in the tumor tissues, showing strong fluorescence staining (Figure 6B).

**Figure 7:**
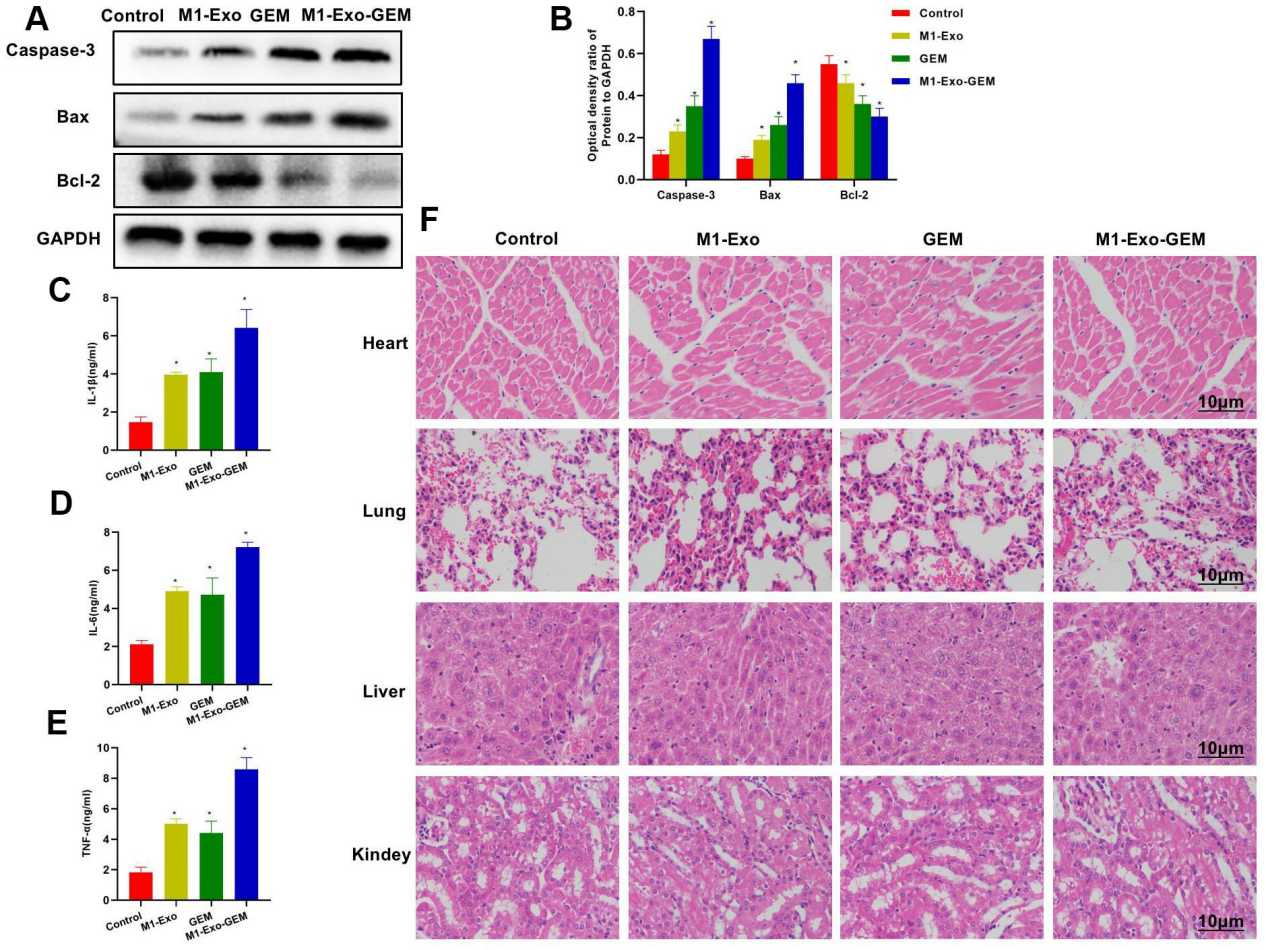
**Effect of M1-Exo-GEM on tumor apoptosis in tumor-bearing mice.** (**A**, **B**) In the protein assay, Bcl-2 was overexpressed in the Control group, while Caspase-3 and Bax were under expressed. GEM and M1-Exo enhanced the expressions of Caspase-3 and Bax and inhibited the Bcl-2expression, suggesting their pro-apoptosis activities. M1-Exo-GEM could further promote the apoptosis signal activation. *P<0.05 vs. Control group. (**B**–**E**) In the detection of tissue inflammatory cytokines, the TNF-α, IL-6 and IL-1β were lowly expressed in the Control group. M1-Exo and GEM enhanced the expressions of these inflammatory cytokines, whose levels were significantly higher than the Control group. M1-Exo-GEM could further up-regulate the inflammatory cytokine levels. *P<0.05 vs. Control group. (**F**) According to the H&E staining results for pathological examination of mouse heart, liver, lung and kidney, M1-Exo-GEM had no toxic effect on other organs. No obvious pathological changes were observed in the organs or tissues, which were in a normal physiological state.

According to protein assay results, GEM and M1-Exo enhanced the expressions of Caspase-3 and Bax and inhibited the Bcl-2expression, suggesting their pro-apoptosis activities. M1-Exo-GEM could further promote the apoptosis signal activation (Figure 7A, 7B). In the inflammatory cytokine detection, the TNF-α, IL-6 and IL-1β were lowly expressed in the Control group. M1-Exo and GEM enhanced the expressions of these inflammatory cytokines, whose levels were significantly higher than the Control group. M1-Exo-GEM could further elevate the inflammatory cytokine levels (Figure 7C–7E). We examined the heart, liver, lung and kidney of mice, and found no obvious tissue lesions in other visceral tissues upon H&E staining, indicating the indistinct toxicity of M1-Exo-GEM to other organs (Figure 7F).

## DISCUSSION

Macrophages, as the major cellular components of the innate immune system, play key roles in inflammation regulation, phagocytosis, tissue remodeling, metabolism and proliferation [12]. As is well acknowledged, nanoparticles are internalized by tumor-associated macrophages in the tumor microenvironment. However, the M1 and M2 macrophages, which differ in phenotype, exert the opposite effects in the body. M1 macrophages can secrete pro-inflammatory cytokines to inhibit tumor growth [13], while M2 macrophages can facilitate tumor growth and metastasis [14]. In tumor-bearing mouse experiments, IFN-γ could induce polarization of resting macrophages toward the pro-inflammatory and tumor cytotoxic M1 phenotype, thereby activating anti-tumor immunity and regulating tumor microenvironment, which exhibited an anti-tumor effect [15]. Activated M1 macrophages were also capable of secreting various inflammatory cytokines (e.g. IL-1β, TNF-γ and IL-6) to induce resistance to intracellular parasites and tumors [16]. Research has shown that the differential regulation of chemokine system could integrate polarized macrophages into pathways that were responsible for tumor resistance, immunoregulation, as well as tissue repair and remodeling [17]. Exos have the characteristics of parent cells, whose roles in intercellular communication and signal transduction have attracted growing attention [18, 19]. Wan et al. found that Exos from tumor tissues could regulate tumor immune response, accelerate tumor metastasis, and induce development of tumor cell resistance to chemotherapeutics [20]. Exos isolated from milk possess an intrinsic anti-tumor activity, while Exos derived from natural killer cells have a cytotoxic effect against melanoma cells *in vitro* [21, 22]. In Exos administration experimentation on tumor-bearing mice, Exos were found to prominently inhibit the tumor growth *in vivo* [23]. Additionally, Exos derived from dendritic cells could induce anti-tumor immune responses both *in vitro* and *in vivo* [24]. Our experiments used the M1-Exos secreted by IFN-γ-induced M1 macrophages, which showed that M1-Exos could activate the apoptotic pathways, showing consistency with the previously reported effect of LPS-induced Exos derived from macrophages. GEM, which represents an important class of anti-tumor drugs, plays a vital role in the treatment of various malignancies. However, its intravesical perfusion yields short efficacy duration and great disparity in outcome. In recent years, Exos, a naturally-derived nanoscale drug carrier, has been used for delivering multiple chemotherapeutics to specific tissues and cells in the body, especially in tumor tissues. Owing to excellent compatibility, Exos have been extensively studied as a drug carrier, which can contain water-soluble and fat-soluble drugs concurrently [5]. Besides, M1-Exos itself can activate immune cells to release anti-tumor cytokines, which is suitable as a carrier for GEM.

In this study, we successfully prepared an M1-Exo-GEM delivery system. During the entrapment process, GEM was embedded in the Exos by ultrasonication, and the Exo membranes were restored to become a drug carrier. We also further evaluated the surface proteins, morphology and electric potential concerning M1-Exo-GEM system. The drug loading process did not affect the protein abundance of Exos. TEM revealed that the morphology and structure of Exos remained basically unchanged after drug loading. Thus, the drug loading process produced unobvious impact on the morphology, structure or other properties of Exos. All the characteristics of Exos were retained after drug loading.

*In vitro* experimentation demonstrated that compared to free GEM, the M1-Exo-GEM system exhibited a higher inhibitory rate against bladder cancer MB49 cells at the same GEM doses, and the relevant mechanism of action might be that the phospholipid bilayer of Exos could directly target fused cells, thereby increasing the cellular internalization of gemcitabine [25]. FCM also revealed that M1-Exo-GEM was more cytotoxic than M1-Exo and GEM. In the mechanism research, we found that M1-Exo-GEM could promote apoptotic signals at a high level, as manifested by significantly up-regulated expressions of Bax and Caspase-3, while suppressing the Bcl-2 expression. In the *in vivo* experimentation, we established a tumor-bearing mouse model for investigating the therapeutic effects of M1-Exo-GEM system through tail vein administration. Compared to the free GEM group, the mice in the M1-Exo-GEM group exhibited the smallest tumor volume, and the tumor volume in the M1-Exos group was also suppressed. This is attributed to the ability of M1-Exos to activate M1 polarization of macrophages *in vivo*, thereby promoting the inflammatory cytokine release. Proteins were extracted from tumor tissues of mice in each group. The Western Blotting results agreed with the *in vitro* experimental findings. GEM itself is a chemotherapeutic drug, which can activate Caspase-3 to induce apoptosis of tumor cells. In the M1-Exos group, the Caspase-3 expression was also up-regulated as compared to the control group. Taken together with *in vitro* experiments, M1-Exos can promote the macrophage release of tumor necrosis factor TNF-α, which binds to the corresponding receptors of tumor cells to activate the downstream Caspase-3. Finally, we also verified that M1-Exo-GEM has no obvious toxicity to normal organs.

## CONCLUSIONS

In this study, GEM, which is widely used in clinical practice, is used as the model drug. Meanwhile, Exos are extracted from activated M1 macrophages by classic ultracentrifugation to serve as the GEM delivery system. The M1-Exo-GEM is fabricated by ultrasonication technique to construct a delivery system, where natural carrier contains chemotherapeutic drug. Our results show that M1-Exo-GEM can exert tumor cytotoxicity more efficiently, whose anti-bladder cancer effect is superior to GEM monotherapy. Its action is associated with the release of inflammatory cytokines and the synergistic drug toxicity.

## References

[1] Hessvik NP, Llorente A. Current knowledge on exosome biogenesis and release. Cell Mol Life Sci. 2018; 75:193–208. 10.1007/s00018-017-2595-9 28733901 PMC5756260

[2] Syn NL, Wang L, Chow EK, Lim CT, Goh BC. Exosomes in Cancer Nanomedicine and Immunotherapy: Prospects and Challenges. Trends Biotechnol. 2017; 35:665–76. 10.1016/j.tibtech.2017.03.004 28365132

[3] Liu C, Guo J, Tian F, Yang N, Yan F, Ding Y, Wei J, Hu G, Nie G, Sun J. Field-Free Isolation of Exosomes from Extracellular Vesicles by Microfluidic Viscoelastic Flows. ACS Nano. 2017; 11:6968–76. 10.1021/acsnano.7b02277 28679045

[4] Crivelli B, Chlapanidas T, Perteghella S, Lucarelli E, Pascucci L, Brini AT, Ferrero I, Marazzi M, Pessina A, Torre ML, and Italian Mesenchymal Stem Cell Group (GISM). Mesenchymal stem/stromal cell extracellular vesicles: From active principle to next generation drug delivery system. J Control Release. 2017; 262:104–17. 10.1016/j.jconrel.2017.07.023 28736264

[5] Zhang W, Yu ZL, Wu M, Ren JG, Xia HF, Sa GL, Zhu JY, Pang DW, Zhao YF, Chen G. Magnetic and Folate Functionalization Enables Rapid Isolation and Enhanced Tumor-Targeting of Cell-Derived Microvesicles. ACS Nano. 2017; 11:277–90. 10.1021/acsnano.6b05630 28005331

[6] Jiang XC, Gao JQ. Exosomes as novel bio-carriers for gene and drug delivery. Int J Pharm. 2017; 521:167–75. 10.1016/j.ijpharm.2017.02.038 28216464

[7] Bellavia D, Raimondo S, Calabrese G, Forte S, Cristaldi M, Patinella A, Memeo L, Manno M, Raccosta S, Diana P, Cirrincione G, Giavaresi G, Monteleone F, et al. Interleukin 3- receptor targeted exosomes inhibit *in vitro* and *in vivo* Chronic Myelogenous Leukemia cell growth. Theranostics. 2017; 7:1333–45. 10.7150/thno.17092 28435469 PMC5399597

[8] Stremersch S, De Smedt SC, Raemdonck K. Therapeutic and diagnostic applications of extracellular vesicles. J Control Release. 2016; 244:167–83. 10.1016/j.jconrel.2016.07.054 27491882

[9] Haney MJ, Klyachko NL, Zhao Y, Gupta R, Plotnikova EG, He Z, Patel T, Piroyan A, Sokolsky M, Kabanov AV, Batrakova EV. Exosomes as drug delivery vehicles for Parkinson’s disease therapy. J Control Release. 2015; 207:18–30. 10.1016/j.jconrel.2015.03.033 25836593 PMC4430381

[10] Morse MA, Garst J, Osada T, Khan S, Hobeika A, Clay TM, Valente N, Shreeniwas R, Sutton MA, Delcayre A, Hsu DH, Le Pecq JB, Lyerly HK. A phase I study of dexosome immunotherapy in patients with advanced non-small cell lung cancer. J Transl Med. 2005; 3:9. 10.1186/1479-5876-3-9 15723705 PMC551593

[11] Besse B, Charrier M, Lapierre V, Dansin E, Lantz O, Planchard D, Le Chevalier T, Livartoski A, Barlesi F, Laplanche A, Ploix S, Vimond N, Peguillet I, et al. Dendritic cell-derived exosomes as maintenance immunotherapy after first line chemotherapy in NSCLC. Oncoimmunology. 2015; 5:e1071008. 10.1080/2162402X.2015.1071008 27141373 PMC4839329

[12] Zanganeh S, Hutter G, Spitler R, Lenkov O, Mahmoudi M, Shaw A, Pajarinen JS, Nejadnik H, Goodman S, Moseley M, Coussens LM, Daldrup-Link HE. Iron oxide nanoparticles inhibit tumour growth by inducing pro-inflammatory macrophage polarization in tumour tissues. Nat Nanotechnol. 2016; 11:986–94. 10.1038/nnano.2016.168 27668795 PMC5198777

[13] Daldrup-Link HE, Golovko D, Ruffell B, Denardo DG, Castaneda R, Ansari C, Rao J, Tikhomirov GA, Wendland MF, Corot C, Coussens LM. MRI of tumor-associated macrophages with clinically applicable iron oxide nanoparticles. Clin Cancer Res. 2011; 17:5695–704. 10.1158/1078-0432.CCR-10-3420 21791632 PMC3166957

[14] Zhao G, Rodriguez BL. Molecular targeting of liposomal nanoparticles to tumor microenvironment. Int J Nanomedicine. 2013; 8:61–71. 10.2147/IJN.S37859 23293520 PMC3534304

[15] Cao Q, Yan X, Chen K, Huang Q, Melancon MP, Lopez G, Cheng Z, Li C. Macrophages as a potential tumor-microenvironment target for noninvasive imaging of early response to anticancer therapy. Biomaterials. 2018; 152:63–76. 10.1016/j.biomaterials.2017.10.036 29111494 PMC5693615

[16] Duluc D, Corvaisier M, Blanchard S, Catala L, Descamps P, Gamelin E, Ponsoda S, Delneste Y, Hebbar M, Jeannin P. Interferon-gamma reverses the immunosuppressive and protumoral properties and prevents the generation of human tumor-associated macrophages. Int J Cancer. 2009; 125:367–73. 10.1002/ijc.24401 19378341

[17] Mantovani A, Sica A, Sozzani S, Allavena P, Vecchi A, Locati M. The chemokine system in diverse forms of macrophage activation and polarization. Trends Immunol. 2004; 25:677–86. 10.1016/j.it.2004.09.015 15530839

[18] Théry C, Ostrowski M, Segura E. Membrane vesicles as conveyors of immune responses. Nat Rev Immunol. 2009; 9:581–93. 10.1038/nri2567 19498381

[19] Wan Y, Wang L, Zhu C, Zheng Q, Wang G, Tong J, Fang Y, Xia Y, Cheng G, He X, Zheng SY. Aptamer-Conjugated Extracellular Nanovesicles for Targeted Drug Delivery. Cancer Res. 2018; 78:798–808. 10.1158/0008-5472.CAN-17-2880 29217761 PMC5811376

[20] An T, Qin S, Xu Y, Tang Y, Huang Y, Situ B, Inal JM, Zheng L. Exosomes serve as tumour markers for personalized diagnostics owing to their important role in cancer metastasis. J Extracell Vesicles. 2015; 4:27522. 10.3402/jev.v4.27522 26095380 PMC4475684

[21] Munagala R, Aqil F, Jeyabalan J, Agrawal AK, Mudd AM, Kyakulaga AH, Singh IP, Vadhanam MV, Gupta RC. Exosomal formulation of anthocyanidins against multiple cancer types. Cancer Lett. 2017; 393:94–102. 10.1016/j.canlet.2017.02.004 28202351 PMC5837866

[22] Munagala R, Aqil F, Jeyabalan J, Gupta RC. Bovine milk-derived exosomes for drug delivery. Cancer Lett. 2016; 371:48–61. 10.1016/j.canlet.2015.10.020 26604130 PMC4706492

[23] Zhu L, Kalimuthu S, Gangadaran P, Oh JM, Lee HW, Baek SH, Jeong SY, Lee SW, Lee J, Ahn BC. Exosomes Derived From Natural Killer Cells Exert Therapeutic Effect in Melanoma. Theranostics. 2017; 7:2732–45. 10.7150/thno.18752 28819459 PMC5558565

[24] Chen S, Lv M, Fang S, Ye W, Gao Y, Xu Y. Poly(I:C) enhanced anti-cervical cancer immunities induced by dendritic cells-derived exosomes. Int J Biol Macromol. 2018; 113:1182–7. 10.1016/j.ijbiomac.2018.02.034 29427678

[25] Tian Y, Li S, Song J, Ji T, Zhu M, Anderson GJ, Wei J, Nie G. A doxorubicin delivery platform using engineered natural membrane vesicle exosomes for targeted tumor therapy. Biomaterials. 2014; 35:2383–90. 10.1016/j.biomaterials.2013.11.083 24345736

